# The relationship between fear of cancer recurrence and posttraumatic growth: a meta-analysis

**DOI:** 10.3389/fpsyg.2024.1373102

**Published:** 2024-05-30

**Authors:** Lianqi Gu, Chen Shen, Linlin Zhao, Na Li, Rao Wang, Lili Dai, Zhiping Chu

**Affiliations:** ^1^School of Nursing, Nanjing Medical University, Nanjing, China; ^2^The Second Affiliated Hospital of Nanjing Medical University, Nanjing, China

**Keywords:** posttraumatic growth, fear of cancer recurrence, cancer, meta-analysis, review

## Abstract

**Background:**

Theoretically, stress is positively correlated with posttraumatic growth (PTG). However, evidence for a correlation between fear of cancer recurrence (FCR), a cancer-specific stressor, and PTG is mixed. The present study aimed to systematically investigate the overall effect size between the two and to explore moderators that may influence this relationship.

**Methods:**

From the earliest available date to October 2023, a comprehensive search was conducted in seven databases. Correlation coefficients (r) were calculated using Stata software. Publication type, continent, trauma role, gender, FCR measurements, PTG measurements, sample size, age, and time since diagnosis were used to examine moderating effects. The National Heart, Lung, and Blood Institute’s (NHLBI) assessment tool was used to evaluate study quality.

**Results:**

A total of 14 studies, involving 17 samples and 3,701 participants, were included. The studies found a small association between FCR and PTG (*r* = 0.161, 95% CI: 0.070–0.249, *p* < 0.01) and large heterogeneity (*I^2^* = 85.5%). The strength of the association varied according to the publication type and FCR measurement.

**Conclusion:**

The current review suggests a small but significant positive correlation between FCR and PTG. Future studies would benefit from exploring additional moderators and the use of standardized, validated FCR measurement tools.

**Systematic review registration:**

PROSPERO, identifier CRD42023460407.

## Introduction

According to GLOBOCAN 2020 statistics, the global cancer burden has risen to 19.3 million new cases and about 10 million deaths (Sung et al., 2021). Due to the unpredictable and incurable nature of cancer, the diagnosis as well as the subsequent treatment can be extremely stressful for the patient and even the entire family (De Padova et al., 2021). In the fourth edition of the Diagnostic and Statistical Manual of Mental Disorders (DSM-IV), cancer serves as a traumatic stressor that can induce serious psychological problems such as post-traumatic stress disorder (PTSD; Leano et al., 2019). With the development of positive psychology, however, it has been found that coping with and dealing with the traumatic experience of cancer may also have positive outcomes. For example, some survivors and their caregivers report positive mental, emotional, cognitive, and behavioral responses following a cancer event (Kim et al., 2007; Li and Loke, 2013; Trevino et al., 2016; Drageset et al., 2020).

The positive psychological changes perceived by individuals in the process of trauma repair are often referred to as post-traumatic growth (PTG; Tedeschi and Calhoun, 1996). In the field of cancer research, studies have shown that PTG can promote the proliferation of white blood cells and lymphocytes and have a positive impact on the body’s immune function (McGregor et al., 2004; Dunigan et al., 2007). In addition, PTG can alleviate patients’ psychological distress, enhance well-being, and improve the quality of life in a disease-bearing state (Husson et al., 2017; Onyedibe et al., 2023). Given its potential benefits, a number of psychosocial interventions have emerged to promote PTG. While these have been shown to be effective overall, many studies still focus on PTG as a secondary outcome to alleviate anxiety and depression (Roepke, 2015; Vrontaras et al., 2023). As cancer is more of a chronic trauma, the development and impact of PTG caused by cancer may differ from other traumas (Menger et al., 2021). Therefore, identifying specific trauma factors associated with cancer is essential to facilitate the use of PTG in therapy.

Co-word analysis of PTG revealed that anxiety and depression emotional responses after negative life events were the primary research hotspots (Yang et al., 2023). However, as early as 2004, a large-sample study pointed out that the most prominent psychological problem for the entire cancer population is actually the fear of cancer recurrence (FCR; Herschbach et al., 2004). FCR refers to a patient’s fear or concern that the cancer may recur or progress (Lebel et al., 2016). Moderate fear facilitates patient monitoring for signs of relapse and promotes active treatment and healthy behaviors (Hawkins et al., 2010; Cincidda et al., 2022). In contrast, excessive fear not only causes negative coping (Oztas et al., 2022), exacerbates psychological distress, and reduces quality of life (Cincidda et al., 2022), but may also increase the incidence of adverse drug reactions (Lu et al., 2023), prompting patients to over-seek medical care and increasing health care costs (Otto et al., 2018). In the long-term follow-up of cancer patients, dealing with FCR is one of the most frequently mentioned unmet needs of many patients after surgery (Dahl et al., 2013; Adashek et al., 2022). Similarly, it is one of the issues that healthcare professionals tend to overlook (Armes et al., 2009).

Considering the prevalence and persistence of FCR, it seems reasonable to understand the course of cancer-related PTG development from that perspective. This can be supported by relevant theoretical models. Tedeschi and Calhoun’s functional descriptive model states that growth is built on distress and that sufficiently intense distress can facilitate cognitive processing and ultimately PTG in individuals (Tedeschi and Calhoun, 2004). The disturbing thoughts of recurrence are similar to intrusive thoughts and may reorganize one’s perception to promote the development of PTG. Several empirical studies have found a positive relationship between FCR and PTG (McDonough et al., 2014; Kuswanto et al., 2020). However, there are also inconsistent findings, such as a possible negative correlation or no significant association between the two (Jones et al., 2017; Darabos et al., 2021; Nik Jaafar et al., 2022).

The existence of conflicting results in multiple studies has to make us question the reliability of the studies in question. And it is by increasing the sample size that meta-analysis improves the certainty of the findings (Borenstein et al., 2021). Therefore, our primary objective in conducting the meta-analysis was to investigate the overall effect size of the association between FCR and PTG across different studies. Additionally, as a secondary objective, we investigated potential moderators that may influence the strength of the association, including sample size, age, time since diagnosis, publication type, continent, FCR and PTG measurement tools, trauma roles, and gender. These factors were selected on the basis of previous studies, and the vast majority of them are thought to modulate the relationship between PTG or FCR and associates (Marziliano et al., 2020; Ning et al., 2023; Wan et al., 2023). In addition to potentially helping to explain the inconsistency of the findings, these factors have the promise of providing a basis for the proper view of FCR to promote cancer-associated PTG in the future.

## Methods

This review was conducted following the Preferred Reporting Items for Systematic Reviews and Meta-Analyses (PRISMA) 2020 guidelines and has been registered in the International Prospective Register of Systematic Reviews (PROSPERO) under the number CRD42023460407.

### Search strategy

Systematic searches were performed in seven databases, including Web of Science, PubMed, Cochrane Library, Embase, CINAHL, PsycINFO, and MEDLINE. The search strategy was to combine Medical Subject Headings (MeSH) and text words related to concepts such as “post-traumatic growth,” “cancer,” “recurrence,” and “fear” and to ensure adaptation to the characteristics of each database. In addition, we examined the reference lists of the articles to identify other relevant studies. The search window was from the date of creation to October 2023 for each database. The full search strategy can be seen in Additional File 1.

### Inclusion and exclusion criteria

Articles were screened according to the following criteria: (1) The subjects were cancer patients or their primary caregivers. (2) FCR and PTG were assessed using quantitative methods. (3) The correlation coefficient, r, between the overall FCR and the overall PTG was reported, or other statistical data that could be transformed into r were provided. (4) The type of study was a cross-sectional study or a longitudinal study. (5) It was written in English and published as a peer-reviewed journal or doctoral dissertation. (6) When the same dataset contained multiple publications, we selected the study with the most complete data.

### Data extraction

We imported the search results into Endnote X9 and eliminated duplicate literature. Two reviewers (LQG and CS) then independently screened the literature and extracted the data. Any differences that arose were resolved through discussion and negotiation with a third reviewer (ZPC). Literature was screened by reading the title and abstract, and after excluding obviously irrelevant literature, the full text was further reviewed for inclusion. Key data from the eligible literature were extracted into a table prepared by the research team. The extraction included (1) basic information about the study, such as authors, year of publication, country, study design, and type of publication. (2) Basic characteristics of the study population, such as sample size, type of cancer, mean age, and time since diagnosis. (3) The measurement tools used and their reliability. (4) Outcome measure data, i.e., Pearson’s correlation coefficient (r).

### Quality assessment

The methodological quality of the study was assessed by two reviewers (LQG and CS) independently using the National Heart, Lung, and Blood Institute’s (NHLBI) Quality Assessment Tool for Observational Cohort and Cross-Sectional Studies (National Heart, Lung, and Blood Institute, 2014). The tool allows researchers to make judgments about the internal validity of a study in a total of four areas of risk: selection bias, information bias, measurement bias, or confounding bias. As the 12th criterion (i.e., blinding of outcome assessors) was not relevant to any of the included studies, we assessed the remaining 13 criteria. Lower quality scores imply a higher risk of bias. Scoring inconsistencies were dealt with by discussion, and a third reviewer (RW) was available for arbitration.

### Data analysis

The Pearson correlation coefficient was used as the main effect size for the meta-analysis. For those studies that did not conduct correlation analyses but reported other available data on the relationships between variables, we chose to reconvert these data to binary correlations by means of the appropriate formulas. Specifically, for studies that reported only *B* or *β*, we first converted *B* to *β* and then converted *β* to *r* via formula (Peterson and Brown, 2005). In addition, we calculated the size of *r* by sample size and *p*-value (Card, 2012).

Since the correlation coefficient is closely related to the standard error (se), the *r* value was converted to Fisher’s *Z* value for meta-analysis (Tural et al., 2022; Welz et al., 2022). The inverse variance method was utilized in Stata software to derive the summary Fisher’s *Z* value and finally converted to an *r* value for interpretation (Tsiligianni et al., 2011). The transformation formulas and codes involved in this study are listed in Additional File 2. As suggested by Cohen (1988), effect size *r* values of 0.1, 0.3, and 0.5 corresponded to weak, moderate, and strong correlations, respectively.

Cochran’s *Q* test and *I^2^* were used to assess the magnitude of study heterogeneity (Higgins and Thompson, 2002). When the test result was *p* < 0.1 and *I^2^* > 50%, a random effects model was used. Otherwise, a fixed effects model was used. When the studies showed heterogeneity, the source of heterogeneity was further assessed by meta-regression and subgroup analysis. As previously mentioned, in this study, we performed meta-regression analyses on continuous moderators (including sample size, mean age, and time since diagnosis). In addition, subgroup analyses were performed on categorical moderators (including publication type, continent of study, FCR vs. PTG measurement tools, trauma role, and gender).

Finally, sensitivity analysis was performed to examine the stability of the findings. Publication bias was solved by a funnel plot, Begg’s test, and Egger’s test (Begg and Mazumdar, 1994; Egger et al., 1997). All statistical tests involved in this study were two-sided, and *p* < 0.05 was considered to indicate statistical significance.

## Results

### Study characteristics and study quality

**Figure 1:**
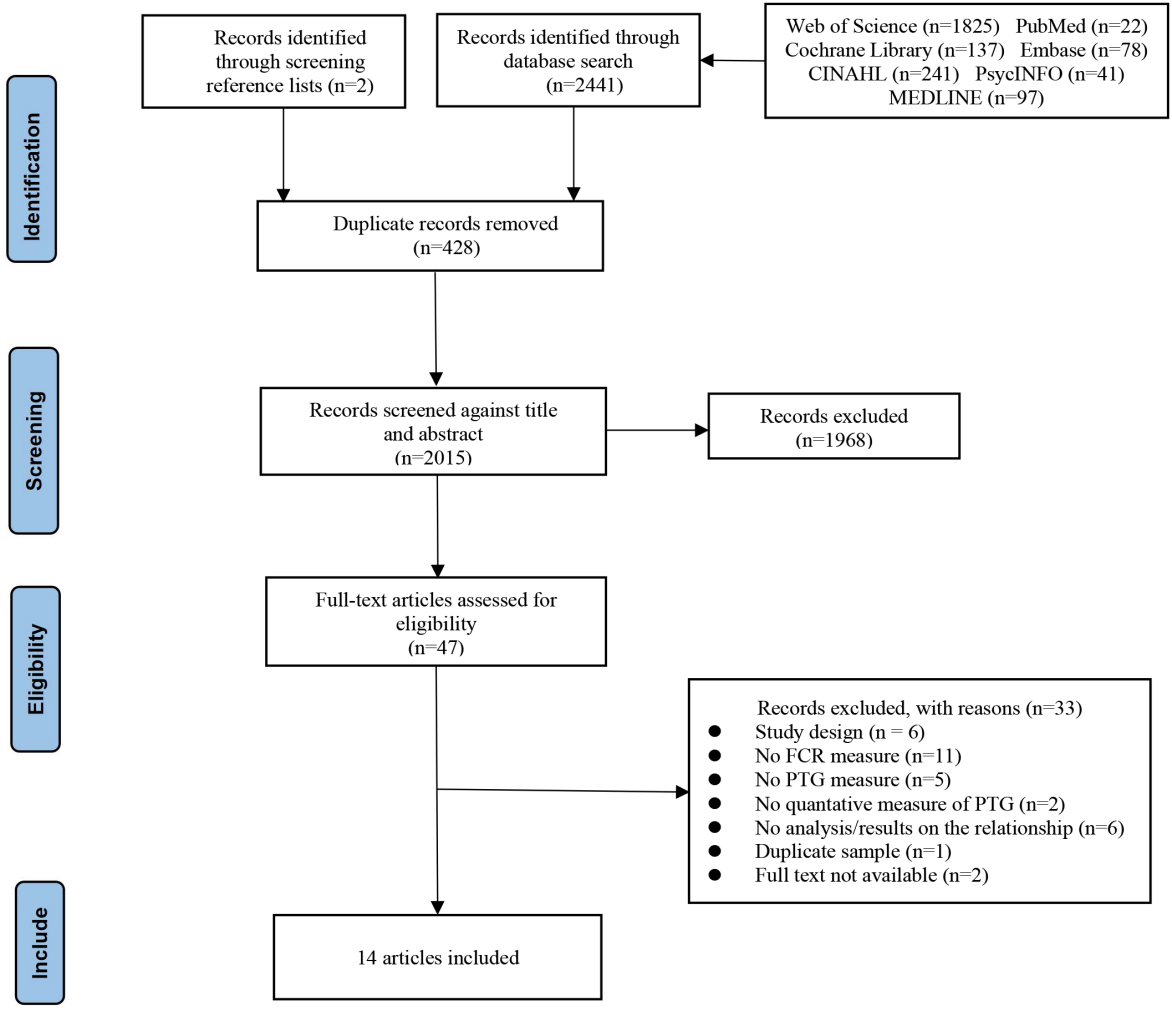
PRISMA flowchart.

An initial 2,443 articles were identified through a systematic search of seven databases and a review of relevant reference lists. After removing duplicates, we read the titles and abstracts of the remaining 2015 articles. Forty-seven articles then required full-text review. Fourteen articles ultimately met the inclusion criteria. Detailed information on study selection is shown in Figure 1.

Table 1 lists the main features of the final 14 articles. These studies were conducted in the Americas, Asia, Europe, and Oceania. This meta-analysis extracted data from 17 studies from the 14 articles, two of which were longitudinal and the rest were cross-sectional in nature. The studies involved a total of 3,701 participants, with sample sizes ranging from 32 to 763, of which 3,224 were female and 477 were male. The mean age of participants ranged from 16.79 to 63.9 years, and 2 studies did not report the mean age. A total of 9 FCR measurement tools and 4 PTG measurement tools were used in the studies. The most commonly used FCR measurement tool was the CARS scale (*n* = 4), while the most commonly used PTG measurement tool was the PTGI scale (*n* = 9). Regarding the calculation of r, data from 12 studies provided r values directly, and data from the remaining 5 studies needed to be transformed by relevant calculation formulas. The included studies were assessed according to the NHLBI Observational Cohort and Cross-Sectional Study Quality Assessment Tool, and ultimately all studies were deemed acceptable (total score ≥ 6), as assessed in detail in Additional File 3.

**Table 1:** Summary of included studies.

Author/ (Year)	Country	Design	Publication type	Sample (male/female)	Cancer Type	Mean Age	Time since diagnosis (Months)	FCR measure	Reliability	PTG measure	Reliability	Main finding
Balfe et al. (2016)	Ireland	C	Journal	194(44/150)	head and neck	N/A	N/A	WOC	unknown	PTGI	Cronbach’s alpha = 0.9	*r* = 0.167
Bower et al. (2005)	USA	C	Journal	763(0/763)	breast	T1: 59 T2: 61.8	T1: 40.8 T2: 74.4	Vulnerability Scale	Cronbach’s alpha = 0.83	Positive Meaning Scale	Cronbach’s alpha = 0.84	T1: *r* = 0.37 T2: *r* = 0.29
Chang et al. (2022)	Taiwan	C	Journal	114(105/9)	head and neck	54.59	4.055	FoP-Q-SF	Cronbach’s alpha = 0.88	PTGI	Cronbach’s alpha = 0.9	*r* = 0.445
Cho et al. (2017)	USA	C	Journal	292(57/235)	mixed	33.32	45.24	ASC	Cronbach’s alpha = 0.81	PTGI	Cronbach’s alpha = 0.95	*r* = 0.03
Darabos et al. (2021)	USA	C	Journal	57(3/54)	mixed	34.68	28.29	CARS	Cronbach’s alpha = 0.96	PTGI-SF	Cronbach’s alpha = 0.88	*r* = 0.02
Jaafar et al. (2022)	Mala1ysia	C	Journal	190(103/87)	head and neck	N/A	N/A	FoP-Q-SF	Cronbach’s alpha = 0.93	PTGI-SF	Cronbach’s alpha = 0.89	*r* = −0.209
Koutna et al. (2021)	CZ	C	Journal	167(86/81)	mixed	16.79	55.92	UCLA_ PTSD	Cronbach’s alpha = 0.90	BFSC	Cronbach’s alpha = 0.90	*r* = 0.197
Kuswanto et al. (2020)	Australia	C	Journal	91(0/91)	breast	50.87	77.27	CARS	Cronbach’s alpha = 0.87	PTGI-SF	Cronbach’s alpha = 0.89	*r* = 0.14
Lo et al. (2023)	Taiwan	C	Journal	120(47/73)	mixed	47.49	N/A	FCRI-SF	Cronbach’s alpha = 0.88	PTGI	Cronbach’s alpha = 0.94	*r* = 0.212
Martens (2017)	USA	C	Dissertation	284(0/284)	breast	35.5	64.8	CARS	Cronbach’s alpha = 0.86	BFS	Cronbach’s alpha = 0.91	*r* = −0.07
McDonough et al. (2014)	USA	C, L^a^	Journal	171(0/171)	breast	55.40	11.37	ASC	Cronbach’s alpha = 0.93	PTGI	Cronbach’s alpha = 0.9	T1: *r* = 0.25 T2: *r* = 0.29
Mell et al. (2022)	USA	L^b^	Journal	154(0/154)	mixed	62.40	N/A	CWS	Cronbach’s alpha = 0.86	PTGI-SF	Cronbach’s alpha = 0.96	*r* = 0.232
Ponto (2009)	USA	C	Dissertation	64(32/32)	ovarian	Survivors: 61.5 Spouses: 63.9	64.9	FRQ	Survivors’ Cronbach’s alpha = 0 0.93 Spouses’ Cronbach’s alpha = 0 0.83	PTGI	Survivors’ Cronbach’s alpha = 0 0.91 Spouses’ Cronbach’s alpha = 0 0.92	Survivors: *r* = 0.02 Spouses: *r* = 0.052
Teixeira da Silva (2016)	USA	C	Dissertation	106(0/106)	breast	58.45	58.32	CARS	Cronbach’s alpha = 0.87	PTGI	Cronbach’s alpha = 0.9	*r* = 0.09

N/A, Not reported. C, cross sectional; L, longitudinal; ^a^FCR measured at Time 1, PTG at Time 2; ^b^PTG measured at Time 1, FCR measured at Time 2; WOC, Worry of Cancer scale; FOP-Q-SF, Fear of Progress Questionnaire-Short Form; ASC, Assessment of Survivor Concerns; CARS, Concerns About Recurrence Scale; UCLA_PTSD, University of California at Los Angeles Posttraumatic Stress Reaction Index; FCRI-SF, Fear of Cancer Recurrence Inventory-Short Form; CWS, Cancer Worry Scale; FRQ, Fear of Recurrence Questionnaire; PTGI, Post traumatic Growth Inventory; PTGI-SF, Short Form of the Posttraumatic Growth Inventory; BFS: Benefit Finding Scale; BFSC, Benefit Finding Scale for Children.

### Meta-analysis

**Figure 2:**
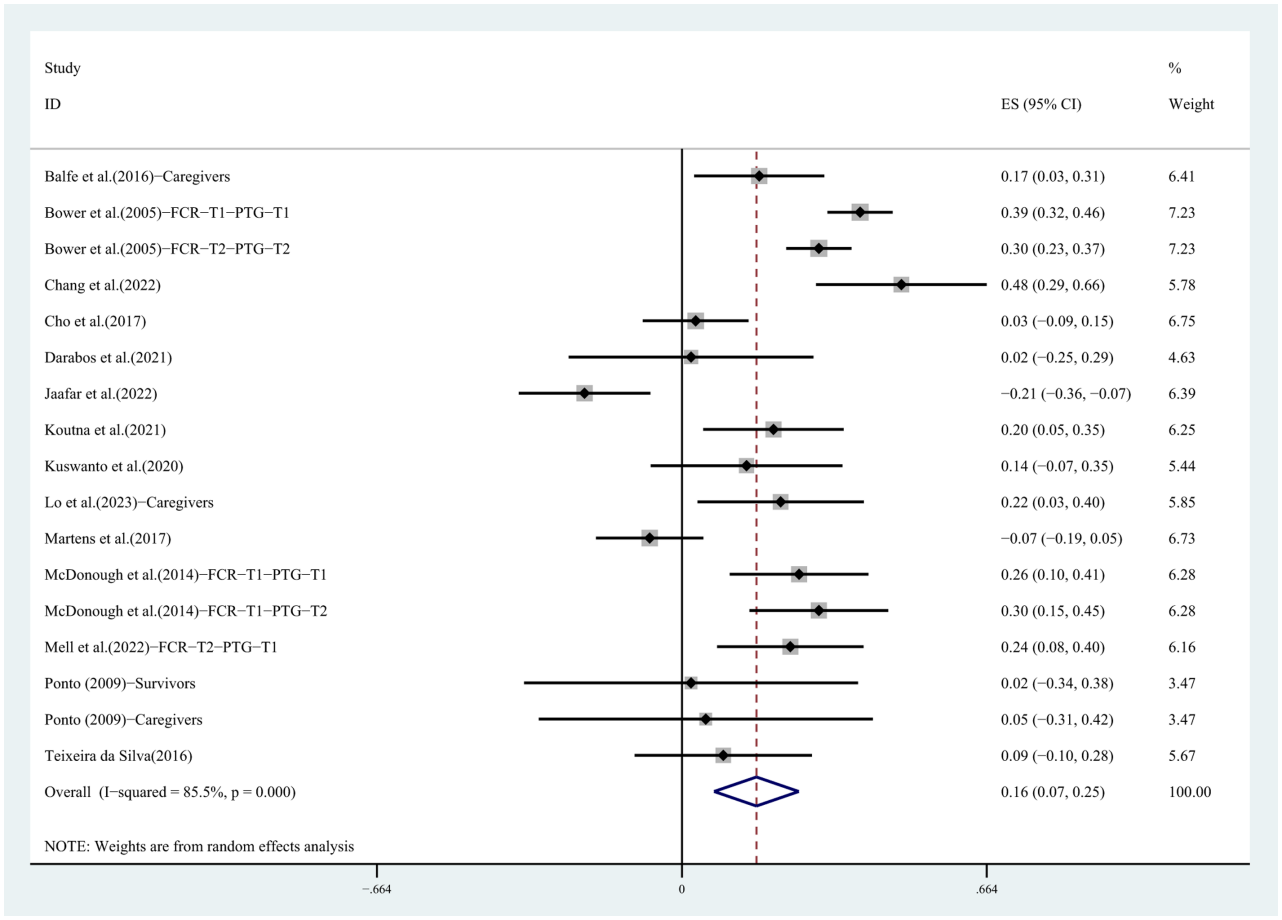
Forest plot for the correlation between FCR and PTG.

Effect sizes from 14 articles (*K* = 17) were synthesized to explore the association between FCR and PTG. Studies reported effect sizes ranging from *r* = −0.209 to *r* = 0.445. One study reported a negative association, 10 studies reported a positive association, and six additional studies had statistically insignificant combined effects. Due to the high degree of heterogeneity across studies (*Q* = 110.62, *p* < 0.001, *I^2^* = 85.5%), we used a random effects model to combine the data. As shown in Figure 2, there was a low positive association between FCR and PTG, *r* = 0.161, 95% CI [0.070, 0.249], *p* < 0.01.

### Moderator analyses

To explore which factors contribute to the correlation between FCR and PTG, a subgroup analysis was conducted, as shown in Table 2. The results indicated that publication type and FCR measurement tools were important moderators. Specifically, journal articles reported a stronger correlation between FCR and PTG (*r* = 0.196) compared with dissertations (*r* = −0.019). With respect to the FCR measurement instrument, the largest effect sizes were reported by studies using the other measures (*r* = 0.246), followed by ASC (*r* = 0.187), then FoP-Q-SF (*r* = 0.130), and the smallest effect sizes were reported by studies using the CARS measure (*r* = 0.020). In contrast, no significant subgroup differences were found in the continents on which the study was conducted, the PTG measurement tool, the traumatized role, or gender factors, although the European group (0%) and the caregiver role group (0%) were not heterogeneous.

**Table 2:** Subgroup analysis of the summary correlation between FCR and PTG.

	Between-group effect (Q_B_)	K	N	Mean r effect size	95% CI for r	Homogeneity test within each group (QW)	I^2^ (%)
Publication type	10.02**						
Journal		13	3,247	0.196	[0.101, 0.287]	82.03***	85.4
Dissertation		4	454	−0.019	[−0.112, 0.074]	2.15	0.0
Continent	0.14						
America		11	2,825	0.164	[0.058, 0.267]	66.97***	85.1
Asia		3	424	0.156	[−0.250, 0.515]	35.65***	94.4
Europe		2	361	0.181	[0.079, 0.279]	0.08	0.0
Oceania		1	91	0.140	[−0.068, 0.336]	0.00	-
FCR measurement	12.25**						
ASC		3	634	0.187	[0.013, 0.349]	9.63**	79.2
CARS		4	538	0.020	[−0.084, 0.124]	3.97	24.5
FoP-Q-SF		2	304	0.130	[−0.498, 0.668]	33.22***	97.0
Others		8	2,225	0.246	[0.173, 0.316]	16.36*	57.2
PTG measurement	1.51						
PTGI		9	1,232	0.192	[0.095, 0.285]	21.89**	63.4
PTGI-SF		4	492	0.044	[−0.185, 0.268]	18.43***	83.7
Others		4	1977	0.206	[0.027, 0.372]	44.59***	93.3
Role	0.03						
Survivors		14	3,355	0.161	[0.056, 0.262]	109.51***	88.1
Caregivers		3	346	0.173	[0.068, 0.274]	0.64	0.0
Gender	1.00						
All female sample		9	2,535	0.198	[0.090, 0.301]	50.51***	84.2
All male sample		1	32	0.052	[−0.302, 0.394]	0.00	-
Combined sample		7	1,134	0.126	[−0.030, 0.275]	39.95***	85.0

*p < 0.05, **p < 0.01, ***p < 0.001.

In contrast, no significant subgroup differences were found in the continents on which the study was conducted, the PTG measurement tool, the role of the traumatized person, and gender, although there was no heterogeneity in the European group (0%) or the caregiver role group (0%).

### Meta-regression

Sample size, age, and time since diagnosis were selected as covariates for meta-regression; however, as shown in Table 3, none of these factors were able to significantly influence the relationship between FCR and PTG (*p* > 0.05).

**Table 3:** Meta-regression analysis of effect sizes between FCR and PTG.

Variables	K	B	SE	95%CI	*t*	*p*
Sample size	17	0.000	0.000	[−0.000, 0.001]	1.39	0.185
Age	15	0.005	0.003	[−0.001, 0.011]	1.89	0.081
Time since diagnosis	13	−0.003	0.002	[−0.007, 0.001]	−1.71	0.115

### Sensitivity analysis

**Figure 3:**
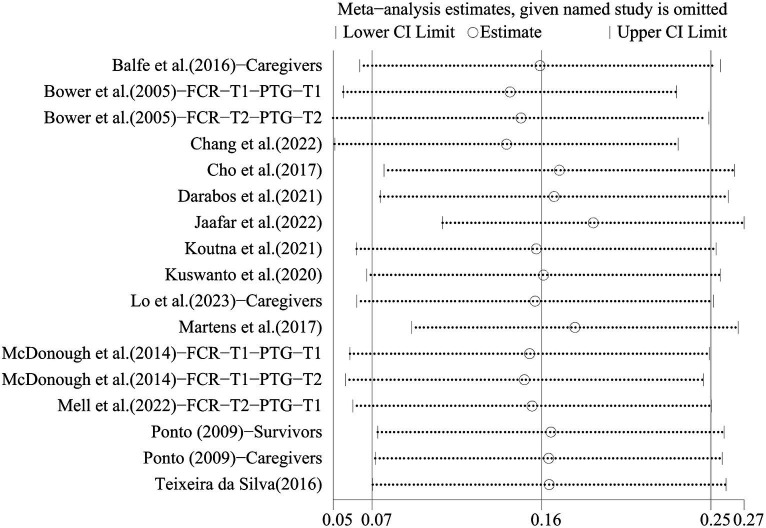
The result of sensitivity analysis.

To assess the robustness of the findings, we sequentially excluded one study and performed a meta-analysis to combine the remaining studies. The results of the sensitivity analysis showed that no individual study had a substantial effect on the pooled effect, i.e., our results were stable (see Figure 3).

### Publication bias

**Figure 4:**
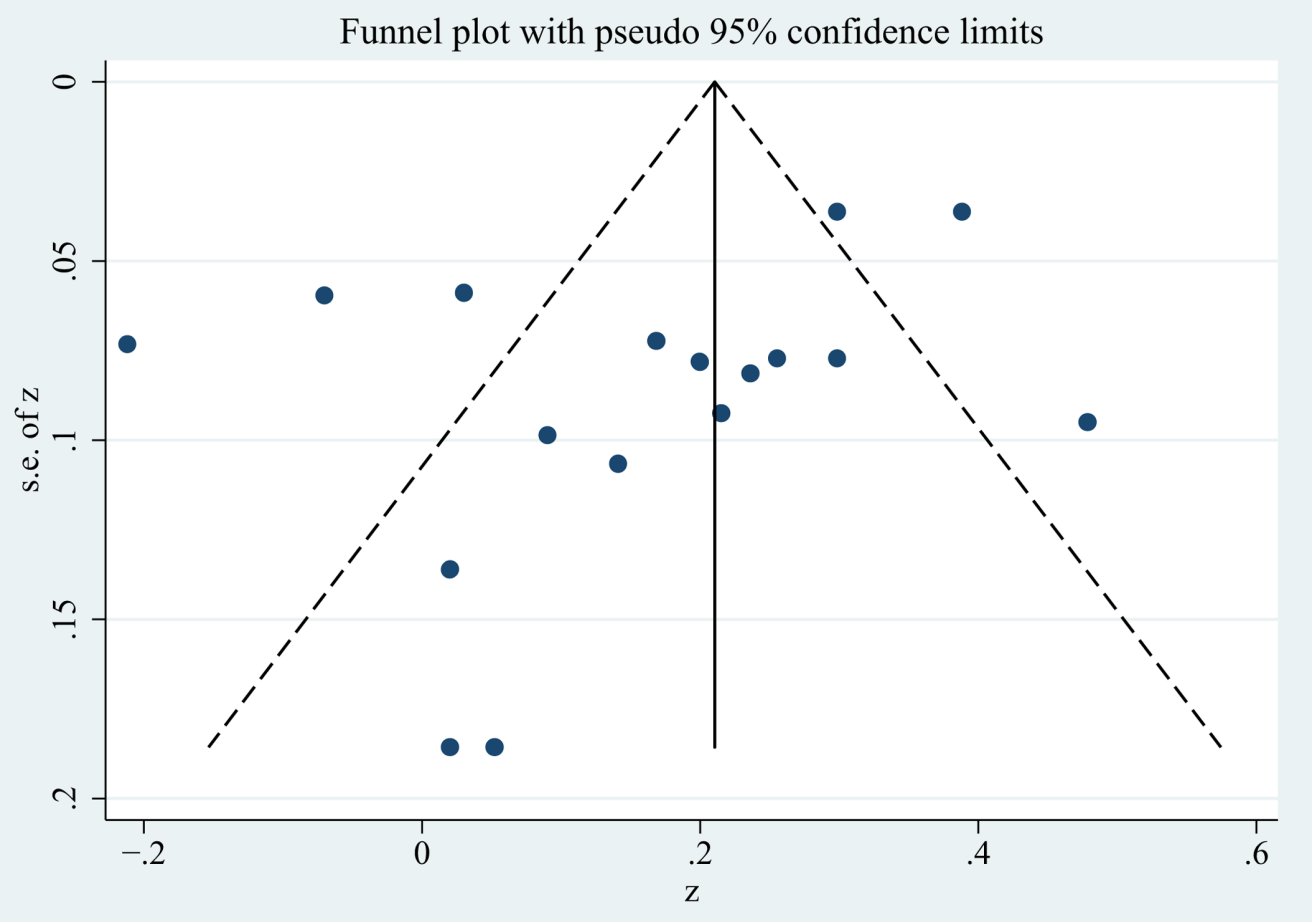
Funnel plots of the meta-analysis.

In the examination of the funnel plot, the 17 effect sizes of the connection between FCR and PTG were basically evenly distributed on both sides of the total effect size (see Figure 4). In addition, the *p* value of Begg’s test was 0.322 and that of Egger’s test was 0.115, both of which were not statistically significant, indicating no significant publication bias.

## Discussion

To our knowledge, this is the first meta-analysis to explore the relationship between FCR and PTG. Although the direction and magnitude of the association were not consistent across studies, the results, which pooled 17 effect sizes from 14 articles, indicated a small positive association between FCR and PTG in general (*r* = 0.161). This is similar to previous studies exploring the relationship between PTSD and PTG in cancer populations (Marziliano et al., 2020). PTG does not counteract the negative effects of cancer, and growth can come with painful experiences. This result corroborates the theory that suggests that distress catalyzes, maintains, and enhances PTG (Tedeschi and Calhoun, 2004).

Due to the significant heterogeneity of the study (*I^2^* = 85.5%), we further conducted a subgroup analysis. The results showed that the continent on which the study was conducted, the PTG measurement tool, the trauma role, and gender were not moderators. Generally speaking, intercontinental differences may reflect cultural differences between countries. Cultural settings may have an impact on psychological changes brought on by traumatic experiences (Calhoun et al., 2010). Previous research on the topic has shown that culture can influence the utilization of social support and coping strategies of individuals experiencing PTSD or PTG (Eissenstat et al., 2022; Hansford and Jobson, 2022; Ning et al., 2023). However, our study did not have similar findings. The results of an international meta-analysis showed no significant intercontinental differences in levels of FCR (Luigjes-Huizer et al., 2022), which may have led to similar levels of cognitive and emotional arousal. Additionally, more than half of the studies took place in the United States, which limits our understanding of the role of culture since it is not possible to further divide these samples based on racial characteristics.

Regarding the measurement of PTG, the most frequently used is PTGI, followed by PTGI-SF, and the others are mainly BFS. Although these instruments differed in terms of the dimensions of growth assessed and the number of questions, they all had good psychometric properties and high interscale correlations (Cann et al., 2010; Jansen et al., 2011). This may have contributed to the nonsignificant moderation effect. It is worth noting that the self-report PTG measure requires patients to make comparisons between existing and past conditions and to determine whether the resulting differences are attributable to the traumatic event (Gower et al., 2022). Thus, when confronted with similar questions or concepts, individuals are likely to lack the time, motivation, or ability to accurately discern the differences between these questions and are thus prone to producing similar responses.

In terms of trauma roles, research has shown that directly traumatized individuals are more likely to develop high levels of PTG than indirectly traumatized individuals (Wu et al., 2019). In other meta-analyses of factors associated with PTG, the caregiver samples were also unique (Shakespeare-Finch and Lurie-Beck, 2014; Ning et al., 2023), which is inconsistent with our findings. The reason for this analysis may be that caregivers are not less stressed by cancer events than the patients themselves (Gregorio et al., 2012). At the same time, it has been shown that caregiver FCR levels are comparable to those of patients and that the two influence each other (Webb et al., 2023). In this context, it is difficult to make a distinction between the strength of the relationship between FCR and PTG by role category.

Gender is an important factor influencing an individual’s understanding of FCR and PTG. A recent meta-analysis showed that female cancer patients tend to report higher levels of FCR (Pang and Humphris, 2021). However, our results show that this does not imply a more favorable development of PTG in women. Previous studies have similarly shown that although women are more likely to derive psychological benefits from social support, women’s social support did not show a stronger association with PTG or PTSS (Allen et al., 2021; Ning et al., 2023). The reason for this analysis may be that the direct effect of gender on PTG is not consistent, and there may also be other mediating factors influencing this association (Ferris and O’Brien, 2022). Future studies should explore more about possible mediating factors to better understand how PTG is promoted in cancer populations of different genders.

The results of meta-regression on the three continuous variables showed that sample size, age, and time since diagnosis were not sources of study heterogeneity. First, to ensure stable correlations, the sample size of studies should be close to 250 (Schönbrodt and Perugini, 2013). In this review, only four studies had sample sizes that met this criterion, pending future analysis of more large-sample data. Second, at the age level, our sample spanned a wide range of ages, ranging from childhood to old age. Yet even so, age did not significantly modulate the relationship between FCR and PTG, which is inconsistent with previous related studies (Vishnevsky et al., 2010; Allen et al., 2022). The limitations of this result must be carefully considered. This is because the direct effect of age on both FCR and PTG is complex. Past systematic evaluations have shown that younger patient ages are associated with elevated FCR levels (Crist and Grunfeld, 2013). However, this age-generated difference may also be influenced by the timing of study assessments (Starreveld et al., 2018). The direct link between age and PTG is even more controversial (Grace et al., 2015; Shand et al., 2015). Finally, in terms of time since diagnosis, recurrence can pose a threat to patients at all stages of survival, and the resulting FCR can become a constant nuisance for them (Bergerot et al., 2020; Schapira et al., 2022). The self-regulation model of illness states that triggers for FCR include both internal and external aspects (Lee-Jones et al., 1997). For one patient, experiencing more physical symptoms, treatment side effects, or physical changes can trigger FCR (Soriano et al., 2019; Brown et al., 2023). For another patient, hearing about someone else’s cancer and being exposed to cancer-related information can trigger FCR (Gill et al., 2004). Thus, at all stages of the cancer trajectory, patients may be dealing with FCR due to various causes, resulting in less variation in the FCR-PTG relationship at different points in the survival trajectory.

Publication type can moderate the relationship between FCR and PTG. In general, studies with significant results are usually prioritized for publication, so gray literature, including dissertations, should be included as much as possible in the meta-analysis (Sterne et al., 2000). As the results of this study show, the relationship between the two reported in journal articles is significant, while the opposite is true for dissertations. It can be hypothesized that the reason for this result is related to the quality of the original studies and the difference in the rigor of the review. It can also be hypothesized that, due to the fact that the number of dissertations included was much smaller than the number of journal articles, the sample sizes of the two groups differed considerably and therefore had an impact on the final results.

The results also showed that the relationship between FCR and PTG was moderated by FCR measurements. The strongest associations were found in studies that used other tools to measure FCR. At present, there is no consensus on the measurement tools of FCR, and a total of six methods are involved in other groups, among which the reliability of the WOC scale is unknown. Examination of instruments from other groups reveals that some instruments are not specifically designed to measure FCR in a particular population. For example, the WOC scale for caregivers is based on the Survivor Scale with the addition of the word “their” and the deletion of entries (Hodges and Humphris, 2009); the UCLA_PTSD Scale is similarly reworded for the relevant entries (Koutná et al., 2021); the Vulnerability Scale and the FRQ Scale include individual vulnerability as well as concerns about health status in addition to measuring FCR components (Northouse, 1981; Bower et al., 2005). Thus, this may have influenced the strength of the relationship between FCR and PTG to some extent. It is important to note that the FCR is a separate, distinct, and multidimensional construct, and most of the existing studies have used short scales to assess the FCR, which may result in a limited number of FCR dimensions being reflected (Simard and Savard, 2009). In addition, the results of the systematic evaluation of FCR measurement tools show that only a relatively small number of tools have comprehensive psychometric validation (Thewes et al., 2012; Webb et al., 2023). Based on this, future studies on this topic need to consider whether the tools can completely reflect the subjects’ FCR, in addition to their role characteristics, when selecting the tools.

### Clinical implications

The current findings suggest that clinicians should also focus on the person’s level of FCR when assessing PTG related to cancer. As has been previously known about FCR, it can either lead to individual maladjustment or promote healthy behaviors (Cincidda et al., 2022). In other words, one patient may wallow in the pain of FCR and fail to experience further growth. Another patient, on the other hand, has similar distress, but the potential for growth cannot be ignored. Considering individual variability, a clinical interview may be able to help physicians better understand each person’s FCR and PTG. If the FCR promotes the individual’s adaptation to the disease, then the clinician needs to tolerate it and encourage them to make positive changes. However, if FCR hinders the individual’s adaptive functioning, then clinicians need to further consider strategies to mitigate FCR.

### Strengths and limitations

This study used meta-analysis to clarify, for the first time, the controversy about the correlation between FCR and PTG in previous empirical studies. All included studies were assessed for methodological quality using the NHLBI’s tools and were of acceptable quality, which increased confidence in the findings. However, some limitations need to be noted. First, we included only English-language publications, which may have led to the omission of some high-quality studies published in other languages. Secondly, the measurement of PTG comes from retrospective and self-reported results. In view of the fact that some scholars have reported the possibility of illusory PTG before (Frazier et al., 2009; Corman et al., 2021), it is necessary to consider the experimental paradigm of PTG measurement in future research. Another limitation is that 76% of the eligible studies had a sample size of less than 250, which suggests that a significant proportion of the coefficients are at risk of being unstable and therefore may not accurately represent the true overall value (Schönbrodt and Perugini, 2013). Last but not least, due to the limited number of included studies, we recommend caution in interpreting our findings, especially in subgroup analyses based on some moderating variables. In the future, more empirical studies could be chosen to further explore the relationship between FCR and PTG.

## Conclusion

In conclusion, there is a small positive correlation between FCR and PTG in cancer patients. The publication type and FCR measurement method moderated the correlation. Future focus should be on exploring possible moderators and using standardized, validated FCR measurement tools to further test the correlation and identify patients in need of PTG interventions.

## Data availability statement

The original contributions presented in the study are included in the article/Supplementary material, further inquiries can be directed to the corresponding author.

## Author contributions

LG: Writing – review & editing, Writing – original draft, Methodology, Formal analysis, Data curation, Conceptualization. CS: Writing – review & editing, Validation, Methodology, Conceptualization. LZ: Writing – review & editing, Software, Data curation. NL: Writing – review & editing, Supervision. RW: Writing – review & editing, Validation, Supervision. LD: Writing – review & editing, Validation, Supervision. ZC: Writing – review & editing, Validation, Supervision, Methodology.
