# Supplementary material for: The relationship between fear of cancer recurrence and posttraumatic growth: a meta-analysis
Source: Front Psychol. 2024 May 30;15:1373102. doi: 10.3389/fpsyg.2024.1373102 (PMC11181912; doi:10.3389/fpsyg.2024.1373102)
Supplement: Supplementary file 1 [file Table_1.DOCX]

| **Web of science** | | Total |
| --- | --- | --- |
| #1 | TS=(Posttraumatic Growth, Psychological or Growth, Psychological Posttraumatic or Psychological Posttraumatic Growth or Post-traumatic Growth, Psychological or Growth, Psychological Post-traumatic or Post traumatic Growth, Psychological or Psychological Post-traumatic Growth or Psychological Post-traumatic Growths or Posttraumatic Growth or Growth, Posttraumatic or PTG or benefit finding or benefit-finding or perceived benefit or perception of benefits or stress related growth or stress-related growth or thriving or adversarial growth) | 661,439 |
| #2 | TS=(Neoplasms or Tumor or Neoplasm or Tumors or Neoplasia or Neoplasias or Cancer or Cancers or Malignant Neoplasm or Malignancy or Malignancies or Malignant Neoplasms or Neoplasm, Malignant or Neoplasms, Malignant) | 7,426,079 |
| #3 | TS=(Recurrence or Recurrences or Recrudescence or Recrudescences or Relapse or Relapses or return* or progress* or spread or coming back) | 4,685,185 |
| #4 | TS=(Fear* or worr* or anxiety or uncertainty or concern*) | 3,016,130 |
| #5 | #1 AND #2 AND #3 AND #4 | 1825 |

| **PubMed** | | Total |
| --- | --- | --- |
| #1 | ("Posttraumatic Growth, Psychological"[Mesh]) OR (Growth, Psychological Posttraumatic[Title/Abstract] OR Psychological Posttraumatic Growth[Title/Abstract] OR Post-traumatic Growth, Psychological[Title/Abstract] OR Growth, Psychological Post-traumatic[Title/Abstract] OR Post traumatic Growth, Psychological[Title/Abstract] OR Psychological Post-traumatic Growth[Title/Abstract] OR Psychological Post-traumatic Growths[Title/Abstract] OR Posttraumatic Growth[Title/Abstract] OR Growth, Posttraumatic[Title/Abstract] OR PTG[Title/Abstract] OR benefit finding[Title/Abstract] OR benefit-finding[Title/Abstract] OR perceived benefit[Title/Abstract] OR perception of benefits[Title/Abstract] OR stress related growth[Title/Abstract] OR stress-related growth[Title/Abstract] OR thriving[Title/Abstract] OR adversarial growth[Title/Abstract]) | 8,485 |
| #2 | ("Neoplasms"[Mesh]) OR ("Tumor"[Title/Abstract] OR "Neoplasm"[Title/Abstract] OR "Tumors"[Title/Abstract] OR "Neoplasia"[Title/Abstract] OR "Neoplasias"[Title/Abstract] OR "Cancer"[Title/Abstract] OR "Cancers"[Title/Abstract] OR "malignant neoplasm"[Title/Abstract] OR "Malignancy"[Title/Abstract] OR "Malignancies"[Title/Abstract] OR "malignant neoplasms"[Title/Abstract] OR "neoplasm malignant"[Title/Abstract] OR "neoplasms malignant"[Title/Abstract]) | 4,904,250 |
| #3 | ("Recurrence"[Mesh]) OR (Recurrences[Title/Abstract] OR Recrudescence[Title/Abstract] OR Recrudescences[Title/Abstract] OR Relapse[Title/Abstract] OR Relapses[Title/Abstract] OR return*[Title/Abstract] OR progress*[Title/Abstract] OR spread[Title/Abstract] OR coming back[Title/Abstract]) | 2,251,099 |
| #4 | Fear*[Title/Abstract] OR worr*[Title/Abstract] OR anxiety[Title/Abstract] OR uncertainty[Title/Abstract] OR concern*[Title/Abstract] | 1,209,620 |
| #5 | #1 AND #2 AND #3 AND #4 | 22 |

| **Cochrane Library** | | Total |
| --- | --- | --- |
| #1 | MeSH descriptor: [Posttraumatic Growth, Psychological] explode all trees |  |
| #2 | (Growth, Psychological Posttraumatic or Psychological Posttraumatic Growth or Post-traumatic Growth, Psychological or Growth, Psychological Post-traumatic or Post traumatic Growth, Psychological or Psychological Post-traumatic Growth or Psychological Post-traumatic Growths or Posttraumatic Growth or Growth, Posttraumatic or PTG or benefit finding or benefit-finding or perceived benefit or perception of benefits or stress related growth or stress-related growth or thriving or adversarial growth):ti,ab,kw |  |
| #3 | #1 OR #2 | 15,788 |
| #4 | MeSH descriptor: [Neoplasms] explode all trees |  |
| #5 | (Tumor or Neoplasm or Tumors or Neoplasia or Neoplasias or Cancer or Cancers or Malignant Neoplasm or Malignancy or Malignancies or Malignant Neoplasms or Neoplasm, Malignant or Neoplasms, Malignant):ti,ab,kw |  |
| #6 | #4 OR #5 | 265,274 |
| #7 | MeSH descriptor: [Recurrence] explode all trees |  |
| #8 | (Recurrences or Recrudescence or Recrudescences or Relapse or Relapses or return* or progress* or spread or coming back):ti,ab,kw |  |
| #9 | #7 OR #8 | 201,976 |
| #10 | (Fear* or worr* or anxiety or uncertainty or concern*):ti,ab,kw | 130,708 |
| #11 | #3 AND #6 AND #9 AND #10 | 137 |

| **Embase** | | Total |
| --- | --- | --- |
| #1 | 'posttraumatic growth (psychology)'/exp |  |
| #2 | 'growth, psychological posttraumatic':ab,ti OR 'psychological posttraumatic growth':ab,ti OR 'post-traumatic growth, psychological':ab,ti OR 'growth, psychological post-traumatic':ab,ti OR 'post traumatic growth, psychological':ab,ti OR 'psychological post-traumatic growth':ab,ti OR 'psychological post-traumatic growths':ab,ti OR 'posttraumatic growth':ab,ti OR 'growth, posttraumatic':ab,ti OR ptg:ab,ti OR 'benefit finding':ab,ti OR 'perceived benefit':ab,ti OR 'perception of benefits':ab,ti OR 'stress related growth':ab,ti OR 'stress-related growth':ab,ti OR thriving:ab,ti OR 'adversarial growth':ab,ti |  |
| #3 | #1 OR #2 | 10,110 |
| #4 | 'neoplasm'/exp |  |
| #5 | tumor:ab,ti OR neoplasm:ab,ti OR tumors:ab,ti OR neoplasia:ab,ti OR neoplasias:ab,ti OR cancer:ab,ti OR cancers:ab,ti OR 'malignant neoplasm':ab,ti OR malignancy:ab,ti OR malignancies:ab,ti OR 'malignant neoplasms':ab,ti OR 'neoplasm, malignant':ab,ti OR 'neoplasms, malignant':ab,ti |  |
| #6 | #4 OR #5 | 6,920,827 |
| #7 | recurrence:ab,ti OR recurrences:ab,ti OR recrudescence:ab,ti OR recrudescences:ab,ti OR relapse:ab,ti OR relapses:ab,ti OR return*:ab,ti OR progress*:ab,ti OR spread:ab,ti OR 'coming back':ab,ti | 3,368,457 |
| #8 | fear*:ab,ti OR worr*:ab,ti OR anxiety:ab,ti OR uncertainty:ab,ti OR concern*:ab,ti | 1,606,106 |
| #9 | #3 AND #6 AND #7 AND #8 | 78 |

| **CINAHL** | | Total |
| --- | --- | --- |
| #1 | MH Posttraumatic Growth, Psychological OR TI ( Growth, Psychological Posttraumatic or Psychological Posttraumatic Growth or Post-traumatic Growth, Psychological or Growth, Psychological Post-traumatic or Post traumatic Growth, Psychological or Psychological Post-traumatic Growth or Psychological Post-traumatic Growths or Posttraumatic Growth or Growth, Posttraumatic or PTG or benefit finding or benefit-finding or perceived benefit or perception of benefits or stress related growth or stress-related growth or thriving or adversarial growth ) OR AB ( Growth, Psychological Posttraumatic or Psychological Posttraumatic Growth or Post-traumatic Growth, Psychological or Growth, Psychological Post-traumatic or Post traumatic Growth, Psychological or Psychological Post-traumatic Growth or Psychological Post-traumatic Growths or Posttraumatic Growth or Growth, Posttraumatic or PTG or benefit finding or benefit-finding or perceived benefit or perception of benefits or stress related growth or stress-related growth or thriving or adversarial growth ) | 67,869 |
| #2 | MH Neoplasms OR TI ( Tumor or Neoplasm or Tumors or Neoplasia or Neoplasias or Cancer or Cancers or Malignant Neoplasm or Malignancy or Malignancies or Malignant Neoplasms or Neoplasm, Malignant or Neoplasms, Malignant ) OR AB ( Tumor or Neoplasm or Tumors or Neoplasia or Neoplasias or Cancer or Cancers or Malignant Neoplasm or Malignancy or Malignancies or Malignant Neoplasms or Neoplasm, Malignant or Neoplasms, Malignant ) | 671,575 |
| #3 | MH Recurrence OR TI ( Recurrences or Recrudescence or Recrudescences or Relapse or Relapses or return* or progress* or spread or coming back ) OR AB ( Recurrences or Recrudescence or Recrudescences or Relapse or Relapses or return* or progress* or spread or coming back ) | 455,153 |
| #4 | TI ( Fear* or worr* or anxiety or uncertainty or concern* ) OR AB ( Fear* or worr* or anxiety or uncertainty or concern* ) | 390,268 |
| #5 | #1 AND #2 AND #3 AND #4 | 241 |

| **PsycINFO** | | Total |
| --- | --- | --- |
| #1 | MA Posttraumatic Growth, Psychological OR TI ( Growth, Psychological Posttraumatic or Psychological Posttraumatic Growth or Post-traumatic Growth, Psychological or Growth, Psychological Post-traumatic or Post traumatic Growth, Psychological or Psychological Post-traumatic Growth or Psychological Post-traumatic Growths or Posttraumatic Growth or Growth, Posttraumatic or PTG or benefit finding or benefit-finding or perceived benefit or perception of benefits or stress related growth or stress-related growth or thriving or adversarial growth ) OR AB ( Growth, Psychological Posttraumatic or Psychological Posttraumatic Growth or Post-traumatic Growth, Psychological or Growth, Psychological Post-traumatic or Post traumatic Growth, Psychological or Psychological Post-traumatic Growth or Psychological Post-traumatic Growths or Posttraumatic Growth or Growth, Posttraumatic or PTG or benefit finding or benefit-finding or perceived benefit or perception of benefits or stress related growth or stress-related growth or thriving or adversarial growth ) | 20,544 |
| #2 | MA Neoplasms OR TI ( Tumor or Neoplasm or Tumors or Neoplasia or Neoplasias or Cancer or Cancers or Malignant Neoplasm or Malignancy or Malignancies or Malignant Neoplasms or Neoplasm, Malignant or Neoplasms, Malignant ) OR AB ( Tumor or Neoplasm or Tumors or Neoplasia or Neoplasias or Cancer or Cancers or Malignant Neoplasm or Malignancy or Malignancies or Malignant Neoplasms or Neoplasm, Malignant or Neoplasms, Malignant ) | 95,076 |
| #3 | MA Recurrence OR TI ( Recurrences or Recrudescence or Recrudescences or Relapse or Relapses or return* or progress* or spread or coming back ) OR AB ( Recurrences or Recrudescence or Recrudescences or Relapse or Relapses or return* or progress* or spread or coming back ) | 288,365 |
| #4 | TI ( Fear* or worr* or anxiety or uncertainty or concern* ) OR AB ( Fear* or worr* or anxiety or uncertainty or concern* ) | 662,990 |
| #5 | #1 AND #2 AND #3 AND #4 | 41 |

| **MEDLINE** | | Total |
| --- | --- | --- |
| #1 | MH Posttraumatic Growth, Psychological OR TI ( Growth, Psychological Posttraumatic or Psychological Posttraumatic Growth or Post-traumatic Growth, Psychological or Growth, Psychological Post-traumatic or Post traumatic Growth, Psychological or Psychological Post-traumatic Growth or Psychological Post-traumatic Growths or Posttraumatic Growth or Growth, Posttraumatic or PTG or benefit finding or benefit-finding or perceived benefit or perception of benefits or stress related growth or stress-related growth or thriving or adversarial growth ) OR AB ( Growth, Psychological Posttraumatic or Psychological Posttraumatic Growth or Post-traumatic Growth, Psychological or Growth, Psychological Post-traumatic or Post traumatic Growth, Psychological or Psychological Post-traumatic Growth or Psychological Post-traumatic Growths or Posttraumatic Growth or Growth, Posttraumatic or PTG or benefit finding or benefit-finding or perceived benefit or perception of benefits or stress related growth or stress-related growth or thriving or adversarial growth ) | 28,971 |
| #2 | MH Neoplasms OR TI ( Tumor or Neoplasm or Tumors or Neoplasia or Neoplasias or Cancer or Cancers or Malignant Neoplasm or Malignancy or Malignancies or Malignant Neoplasms or Neoplasm, Malignant or Neoplasms, Malignant ) OR AB ( Tumor or Neoplasm or Tumors or Neoplasia or Neoplasias or Cancer or Cancers or Malignant Neoplasm or Malignancy or Malignancies or Malignant Neoplasms or Neoplasm, Malignant or Neoplasms, Malignant ) | 3,510,827 |
| #3 | MH Recurrence OR TI ( Recurrences or Recrudescence or Recrudescences or Relapse or Relapses or return* or progress* or spread or coming back ) OR AB ( Recurrences or Recrudescence or Recrudescences or Relapse or Relapses or return* or progress* or spread or coming back ) | 2,476,479 |
| #4 | TI ( Fear* or worr* or anxiety or uncertainty or concern* ) OR AB ( Fear* or worr* or anxiety or uncertainty or concern* ) | 1,213,254 |
| #5 | #1 AND #2 AND #3 AND #4 | 97 |
