# Supplementary material for: The relationship between fear of cancer recurrence and posttraumatic growth: a meta-analysis
Source: Front Psychol. 2024 May 30;15:1373102. doi: 10.3389/fpsyg.2024.1373102 (PMC11181912; doi:10.3389/fpsyg.2024.1373102)
Supplement: Supplementary file 2 [file Table_2.DOCX]

**Arithmetic formulas involved in data extraction**

**Conversion formula of B to β**

β=B*Sx/Sy

Sx: SD of independent variable,

Sy: SD of dependent variable,

B: unstandardized coefficient,

β: standardized coefficient,

**Conversion formula of β to r**

r=β＋0.05λ

r: correlation coefficient

λ is an indicator variable that equals 1 when β is nonnegative and 0 when β is negative

**Conversion formula of Z value of statistical significance to r**

r=$\sqrt{\frac{Z^{2}}{N}}$

N: sample size

Z: Find the standardized normal deviation (i.e., Z score) corresponding to a given two-tailed p based on the exact probability p of the significance test.

**Analytical codes covered in this study**

**Fisher’s Z values (z sez) transformed by r**

generate z = atanh(r)

generate sez= sqrt(1/(n-3))

**Summary r transformed by the overall Fisher’s Z from meta-analyses**

display _newline "Pooled estimate of r = " tanh(r(ES))

display _newline "Lower Limit of 95% CI = " tanh(r(ci_low))

display _newline "Upper Limit of 95% CI = " tanh(r(ci_upp))
