# Supplementary material for: The relationship between fear of cancer recurrence and posttraumatic growth: a meta-analysis
Source: Front Psychol. 2024 May 30;15:1373102. doi: 10.3389/fpsyg.2024.1373102 (PMC11181912; doi:10.3389/fpsyg.2024.1373102)
Supplement: Supplementary file 3 [file Table_3.DOCX]

| **Additional File 3. National Heart, Lung, and Blood Institute’s (NHLBI) Tool Results** |
| --- |

| Author/(Year) | 1 | 2 | 3 | 4 | 5 | 6 | 7 | 8 | 9 | 10 | 11 | 13 | 14 | Total |
| --- | --- | --- | --- | --- | --- | --- | --- | --- | --- | --- | --- | --- | --- | --- |
| Balfe et al. (2016) | Yes | No | Yes | Yes | No | No | No | Yes | No | No | Yes | NA | Yes | 6 |
| Bower et al. (2005) | Yes | Yes | No | Yes | No | Yes | Yes | Yes | Yes | Yes | Yes | No | Yes | 10 |
| Chang et al. (2022) | Yes | Yes | Yes | Yes | No | No | No | Yes | Yes | No | Yes | NA | Yes | 8 |
| Cho et al. (2017) | Yes | No | Yes | No | No | No | No | Yes | Yes | No | Yes | NA | Yes | 6 |
| Darabos et al. (2021) | Yes | Yes | Yes | NR | Yes | No | No | Yes | Yes | No | Yes | NA | Yes | 8 |
| Jaafar et al. (2022) | Yes | Yes | Yes | Yes | Yes | No | No | Yes | Yes | No | Yes | NA | Yes | 9 |
| Koutna et al. (2021) | Yes | Yes | Yes | NR | No | No | No | Yes | Yes | No | Yes | NA | Yes | 7 |
| Kuswanto et al. (2020) | Yes | No | Yes | Yes | No | No | No | Yes | Yes | No | Yes | NA | Yes | 7 |
| Lo et al. (2023) | Yes | Yes | Yes | Yes | No | No | No | Yes | Yes | No | Yes | NA | Yes | 8 |
| Martens (2017) | Yes | Yes | Yes | Yes | Yes | No | No | Yes | Yes | No | Yes | NA | No | 8 |
| McDonough et al. (2014) | Yes | Yes | Yes | Yes | No | Yes | Yes | Yes | Yes | Yes | Yes | Yes | Yes | 12 |
| Mell et al. (2022) | Yes | Yes | Yes | Yes | No | Yes | Yes | Yes | Yes | Yes | Yes | No | Yes | 11 |
| Ponto (2009) | Yes | Yes | Yes | Yes | Yes | No | No | Yes | Yes | No | Yes | NA | No | 8 |
| Teixeira da Silva (2016) | Yes | Yes | Yes | Yes | Yes | No | No | Yes | Yes | No | Yes | NA | Yes | 9 |

*Note.* Item 12 is not reported as it was not relevant for any of the included studies; NA, not applicable; NR, not reported
